# Supplementary material for: Environmental diversity of Candidatus Babelota and their relationships with protists
Source: mSystems. 2025 May 28;10(6):e00261-25. doi: 10.1128/msystems.00261-25 (PMC12172432; doi:10.1128/msystems.00261-25)
Supplement: File S1 — Details on primer and probes design and validation. [file msystems.00261-25-s0001.docx]

Supplementary file 1

Environmental diversity of *Candidatus* Babelota and their relationships with protists

Louis Weisse, Lucile Martin, Bouziane Moumen, Yann Héchard, Vincent Delafont

FISH probe design and validation

Candidate probes were designed in ARB software, coupled with the SILVA database release 128. A subset of candidate division TM6 sequences were selected as a seed for producing probes [1, 2]. The probe design tool in ARB was set up to reject non group hit and candidate probes were filtered to be 18 nt long, with a GC content between 50% to 100%, and a melting temperature between 30 to 100°C. One probe, TM6_681 (5’-GCATTTTACCGCTACTCC-3’) was further selected for showing both perfect match for *Ca.* Babelota sequences, and showing strong mismatches with non-group hots (*i.e.* mismatch positioned on the central part of the probe). Universality and specificity was further tested using Decipher and Testprobe online tools [2, 3]. The probe TM6_681 yields a 78.9% coverage of all Ca. Babelota sequences currently in SILVA database as of January 2025. Probe hybridization efficiency according to formamide concentration was evaluated in silico using mathFISH [4]. Formamide curves were then generated in the lab using a culture of *Vermiphilus* *pyriformis* grown in its natural amoeba host, resulting in an optimal signal intensity at 15% formamide.

qPCR primer design and validation

Primer pairs targeting *Ca.* Babelota were designed based on an alignment of full length 16S rRNA sequences from *Ca.* Babelota, extracted from the SILVA database. A simple alignement of 15 sequences from diverse *Ca.* Babelota was achieved, using muscle v3.8.31 [5]. the alignment was loaded in the web interface of PrimerDesign-M [6]. Multiple primers pairs were designed, producing amplicon from 80 to 200 bp long, flanking each variable region from V1 to V5. Each primer pair was then double-checked for coverage and specificity, using SILVA testprime web tool. From a total of 13 pairs, 2 showed promising features as they covered, in a specific manner, more than 75% of Ca. Babelota sequences. PCR testing of those 2 pairs excluded one which was producing multiple amplicons despite the application of PCR optimization. Thus, one primer pair remained, flanking the V5 16S rRNA variable region: TM6-V5-F2 (5’-GGAGTAGCGGTAAAATGC-3’) and TM6-V5-R4, (5’-CTACCAGGGTATCTAATCC-3’). This pair is predicted to cover 76.8% of all Ca. Babelota sequences from the SILVA database, as of January 2025. qPCR optimizations were achieved on a lightcycler 480 (Roche), and PCR products were also characterized using a bioanalyzer (Agilent). PCR gradient indicated that the optimal hybridization temperature was situated between 59 to 63°C, hence the choice of applying 60°C for all subsequent assays with this primer pair.

**Litterature cited**

1. Ludwig W, Strunk O, Westram R, Richter L, Meier H, Yadhukumar, et al. ARB: a software environment for sequence data. *Nucleic Acids Res* 2004; **32**: 1363–1371.

2. Quast C, Pruesse E, Yilmaz P, Gerken J, Schweer T, Yarza P, et al. The SILVA ribosomal RNA gene database project: improved data processing and web-based tools. *Nucleic Acids Res* 2013; **41**: D590-6.

3. Wright ES, Yilmaz LS, Corcoran AM, Ökten HE, Noguera DR. Automated design of probes for rRNA-targeted fluorescence in situ hybridization reveals the advantages of using dual probes for accurate identification. *Appl Environ Microbiol* 2014; **80**: 5124–5133.

4. Yilmaz LS, Parnerkar S, Noguera DR. MathFISH, a web tool that uses thermodynamics-based Mathematical models for in silico evaluation of oligonucleotide probes for fluorescence in situ hybridization. *Appl Environ Microbiol* 2011; **77**: 1118–1122.

5. Edgar RC. MUSCLE: multiple sequence alignment with high accuracy and high throughput. *Nucleic Acids Res* 2004; **32**: 1792–1797.

6. Yoon H, Leitner T. PrimerDesign-M: a multiple-alignment based multiple-primer design tool for walking across variable genomes. *Bioinformatics* 2015; **31**: 1472–1474.
