# Supplementary material for: Environmental diversity of Candidatus Babelota and their relationships with protists
Source: mSystems. 2025 May 28;10(6):e00261-25. doi: 10.1128/msystems.00261-25 (PMC12172432; doi:10.1128/msystems.00261-25)
Supplement: Figure S1 — Mean abundances of Ca. Babelota detected by quantitative PCR, per sample. [file msystems.00261-25-s0002.pdf]

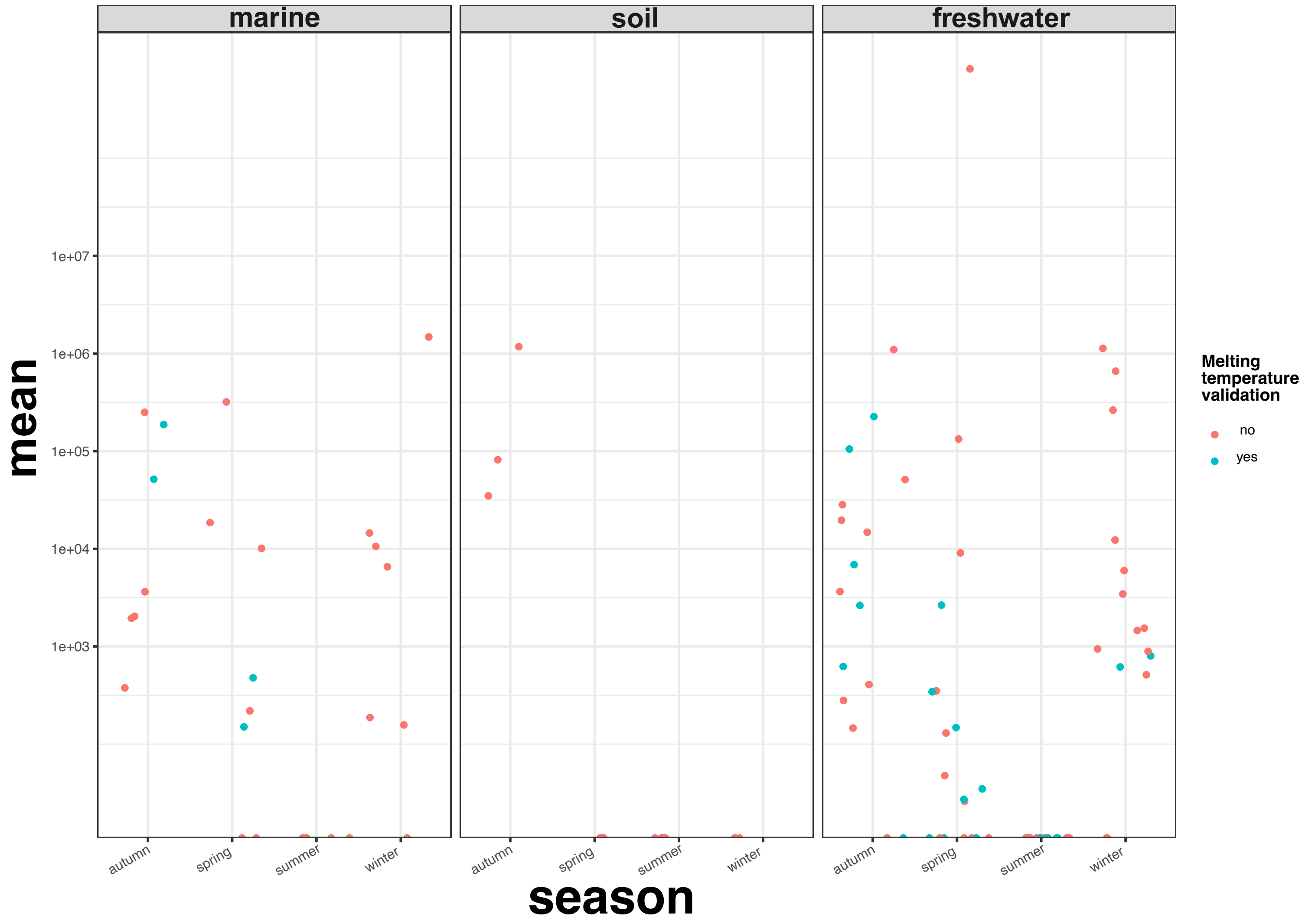

Supplementary Figure 1: Mean abundances of *Ca.* Babelota detected by quantitative PCR, per sample.

Melting temperature validation corresponds to values +/- 0.5°C compared to the standard curve generated with Vermiphilus pyriformis DNA
