# Supplementary material for: Environmental diversity of Candidatus Babelota and their relationships with protists
Source: mSystems. 2025 May 28;10(6):e00261-25. doi: 10.1128/msystems.00261-25 (PMC12172432; doi:10.1128/msystems.00261-25)
Supplement: Figure S2 — Ca. Babelota can be maintained in culture through protist enrichments and are systematically intracellular. [file msystems.00261-25-s0003.pdf]

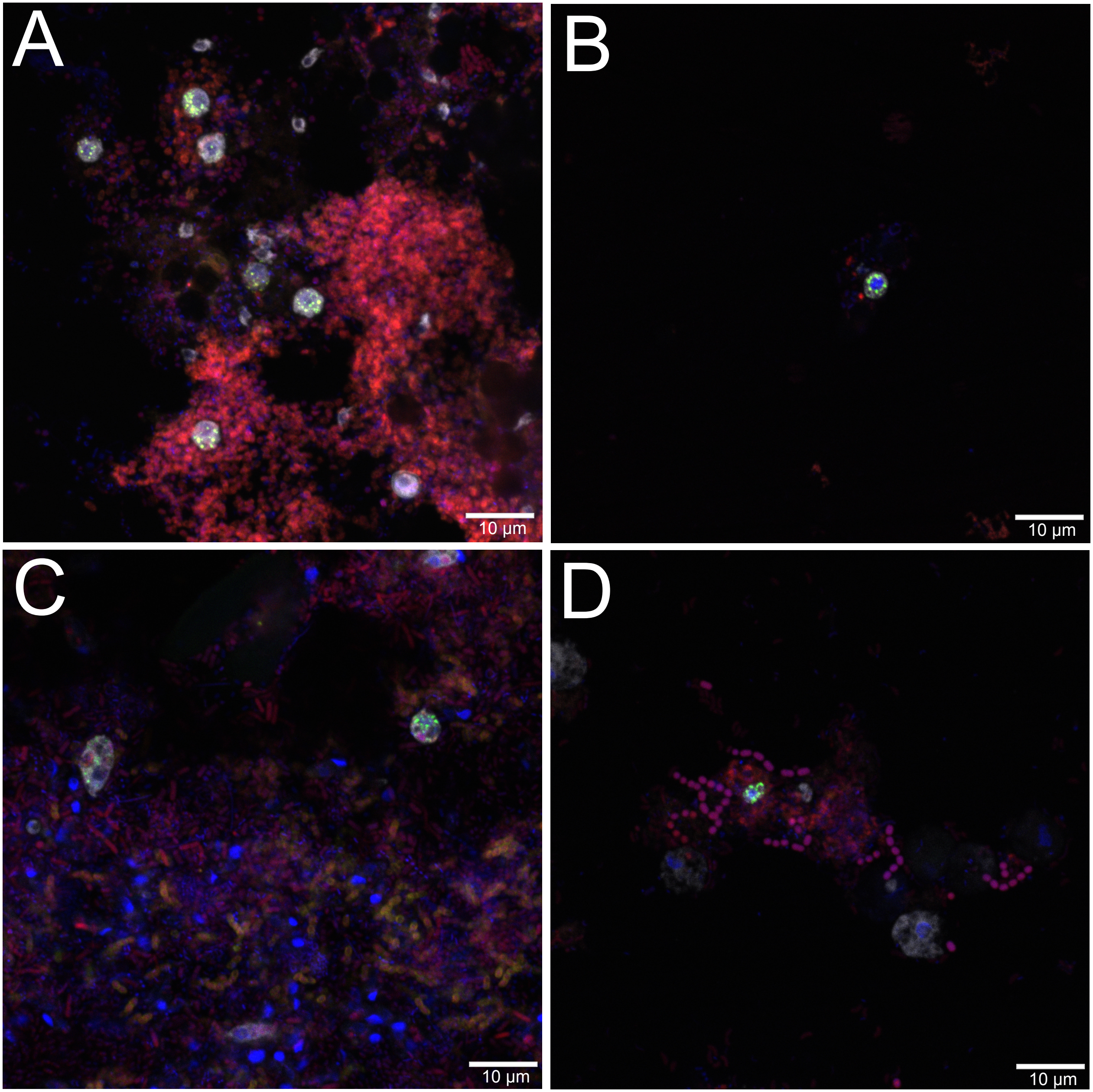

Supplementary Figure 2: *Ca.* Babelota can be maintained in culture through protist enrichments and are systematically intracellular. Fish image of protist enrichments from the first protist enrichment of water from (A) a small pond in a paddock and (B) a pond cover in duckweeb as well as from the third enrichment of water from (C) the paddock or (D) covered in duckeweeb. Green signal corresponds to probe TM6_681 targeting *Ca.* Babelota. Red signal corresponds to the eubacterial probes EUB338-I-III. White signal corresponds to EUK516 probe targeting eukaryotic cells. DNA was labelled using DAPI, shown in blue.
