# Supplementary figures and images for: Environmental diversity of Candidatus Babelota and their relationships with protists
Source: mSystems. 2025 May 28;10(6):e00261-25. doi: 10.1128/msystems.00261-25 (PMC12172432; doi:10.1128/msystems.00261-25)

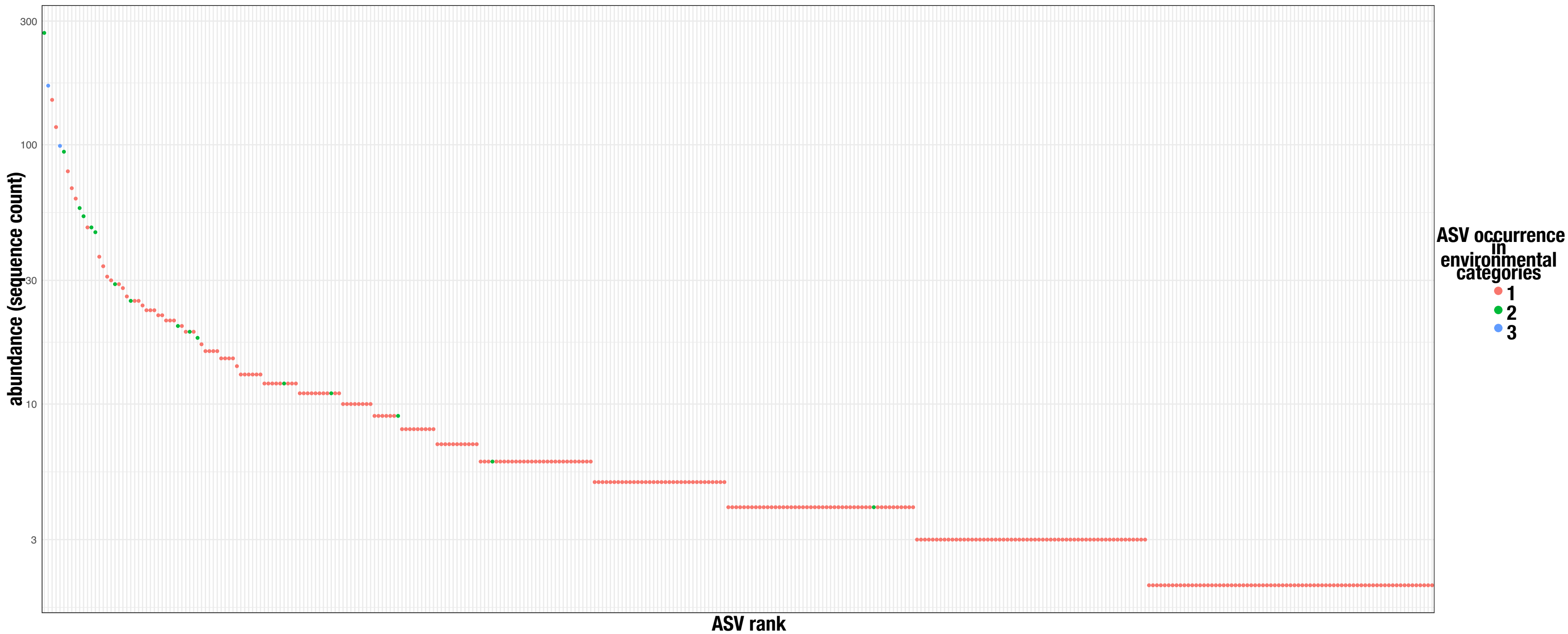

Supplementary Figure 3: Rank abundance of all identified *Ca. Babelota* ASV.

Supplement: Figure S3 — Rank abundance of all identified Ca. Babelota ASV. [file msystems.00261-25-s0004.pdf]
